# Supplementary material for: Reconciling Mining with the Conservation of Cave Biodiversity: A Quantitative Baseline to Help Establish Conservation Priorities
Source: PLoS One. 2016 Dec 20;11(12):e0168348. doi: 10.1371/journal.pone.0168348 (PMC5173368; doi:10.1371/journal.pone.0168348)
Supplement: S1 Dataset — (ZIP) [file pone.0168348.s002.zip › Taxa/Serra Norte/SN_2013/N5S/Lista N5S-636465.pdf]

## CAVIDADE N5S-0063/0064/0065

| Classe | Ordem | Fam/Outros | Gên/Outros | Espécie | S-U | U-S | Zon. |
|---|---|---|---|---|---|---|---|
| Annelida | Oligochaeta | | | jovem | 10 | 9 | E/P |
| Arachnida | Acari | (Oribatida) | | sp.1 | | X | E |
| Arachnida | Acari | (Oribatida) | | sp.12 | X | | E |
| Arachnida | Acari | (Parasitengonina) | | sp.7 | | X | P |
| Arachnida | Acari | Bdellidae | | sp.1 | X | | P |
| Arachnida | Acari | Eupodidae | | sp.1 | | X | E |
| Arachnida | Acari | Ixodidae | *Amblyomma* | sp.1 | X | X | P |
| Arachnida | Acari | Laelapidae | | sp.3 | X | | P |
| Arachnida | Acari | Laelapidae | | sp.41 | X | | P |
| Arachnida | Acari | Rhagidiidae | | sp.5 | | X | P |
| Arachnida | Acari | Sphaerolichidae | | sp.1 | | X | P |
| Arachnida | Acari | Trombiculidae | | sp.39 | | X | P |
| Arachnida | Acari | Veigaiidae | | sp.1 | | X | P |
| Arachnida | Amblypygi | Charinidae | *Charinus* | sp.1 | 1 | 2 | E/P |
| Arachnida | Amblypygi | Phrynidae | *Heterophrynus* | *longicornis* | 14 | 9 | E/P |
| Arachnida | Araneae | Araneidae | *Alpaida* | aff. *negro* | X | | E/P |
| Arachnida | Araneae | Araneidae | *Alpaida* | *antonio* | X | | E/P |
| Arachnida | Araneae | Araneidae | | jovem | X | X | E/P |
| Arachnida | Araneae | Barychelidae | | sp.1 | 2 | 3 | E |
| Arachnida | Araneae | Caponiidae | | sp.1 | X | X | E/P |
| Arachnida | Araneae | Corinnidae | | jovem | 9 | 5 | E/P |
| Arachnida | Araneae | Ctenidae | | jovem | 3 | 5 | E/P |
| Arachnida | Araneae | Drymusidae | *Drymusa* | *spelunca* | X | X | E/P |
| Arachnida | Araneae | Filistatidae | | sp.1 | X | | E |
| Arachnida | Araneae | Linyphiidae | *Tutaibo* | sp.1 | X | | E |
| Arachnida | Araneae | Mysmenidae | *Microdipoena* | sp.1 | X | | E |
| Arachnida | Araneae | Nesticidae | *Nesticus* | sp.2 | X | | P |
| Arachnida | Araneae | Ochyroceratidae | *Ochyrocera* | sp.1 | X | X | E/P |
| Arachnida | Araneae | Ochyroceratidae | *Ochyrocera* | sp.3 | | X | P |
| Arachnida | Araneae | Oonopidae | aff. *Xyccarph* | sp.2 | X | | E |
| Arachnida | Araneae | Oonopidae | aff. *Xyccarph* | sp.3 | X | | E |
| Arachnida | Araneae | Oonopidae | aff. *Xyccarph* | sp.4 | | X | E |
| Arachnida | Araneae | Pholcidae | *Leptopholcus* | sp.1 | X | X | E/P |
| Arachnida | Araneae | Pholcidae | *Mesabolivar* | *aurantiacus* | X | X | E |
| Arachnida | Araneae | Pholcidae | *Mesabolivar* | sp.1 | X | X | E/P |
| Arachnida | Araneae | Pisauridae | | jovem | 1 | 1 | E/P |
| Arachnida | Araneae | Prodidomidae | *Lygromma* | sp.1 | X | | E |
| Arachnida | Araneae | Prodidomidae | *Lygromma* | sp.3 | | X | E |
| Arachnida | Araneae | Salticidae | | sp.1 | | X | E |
| Arachnida | Araneae | Salticidae | | sp.2 | X | | E |
| Arachnida | Araneae | Salticidae | | sp.7 | X | | E |
| Arachnida | Araneae | Salticidae | | sp.9 | X | | E |
| Arachnida | Araneae | Scytodidae | *Scytodes* | sp.1 | 10 | 13 | E |
| Arachnida | Araneae | Tetragnathidae | *Azilia* | *histrio* | | X | E |
| Arachnida | Araneae | Theraphosidae | aff. *Holothele* | sp.1 | 6 | 6 | E |
| Arachnida | Araneae | Theraphosidae | aff. *Holothele* | sp.2 | 1 | | |
| Arachnida | Araneae | Theridiidae | *Theridion* | sp.1 | X | X | E/P |
| Arachnida | Araneae | Theridiidae | *Theridion* | sp.2 | | X | E |
| Arachnida | Araneae | Theridiidae | *Theridion* | sp.4 | X | X | E/P |

| | | | | | | | |
|---|---|---|---|---|---|---|---|
| Arachnida | Araneae | Theridiosomatidae | *Plato* | sp.1 | X | X | E/P |
| Arachnida | Araneae | Uloboridae | | sp.1 | X | | E |
| Arachnida | Araneae | Uloboridae | | sp.2 | X | | P |
| Arachnida | Opiliones | Escadabiidae | | sp.2 | X | X | P |
| Arachnida | Opiliones | Sclerosomatidae | *Geaya* | sp.1 | 1 | | |
| Arachnida | Opiliones | Sclerosomatidae | *Prionostema* | sp.1 | 2 | 2 | E |
| Arachnida | Opiliones | Stygnidae | *Paraphareus* | sp.1 | | X | P |
| Arachnida | Opiliones | Stygnidae | *Protimesius* | *gracilis* | 3 | 1 | P |
| Arachnida | Pseudoscorpiones | Bochicidae | | sp.1 | X | X | P |
| Arachnida | Pseudoscorpiones | Chernetidae | *Spelaeochernes* | sp.1 | X | X | E/P |
| Arachnida | Pseudoscorpiones | Chthoniidae | *Pseudochthonius* | sp.1 | X | X | P |
| Chilopoda | Geophilomorpha | Ballophilidae | *Ityphilus* | sp.1 | | 3 | P |
| Chilopoda | Geophilomorpha | Geophilidae | | sp.2 | 1 | | P |
| Chilopoda | Geophilomorpha | Schendylidae | | sp.1 | | 1 | E |
| Chilopoda | Scolopendromorpha | Scolopendridae | *Otostigmus* | sp.1 | | 1 | E |
| Chilopoda | Scolopendromorpha | Scolopocryptopidae | *Newportia* | sp.1 | 1 | 1 | P |
| Chilopoda | Scutigeromorpha | Pselliodidae | *Sphendononema* | jovem | 2 | | E/P |
| Diplopoda | Polydesmida | Chelodesmidae | | sp.3 | 11 | 9 | P |
| Diplopoda | Polydesmida | Fuhrmannodesmidae | | jovem | X | X | P |
| Diplopoda | Polydesmida | Pyrgodesmidae | | sp.1 | X | X | P |
| Diplopoda | Spirostreptida | Pseudonannolenidae | *Pseudonannolene* | jovem | 1 | | P |
| Entognatha | Collembola | Cyphoderidae | | sp.1 | X | X | E/P |
| Entognatha | Collembola | Entomobryidae | | sp.1 | X | | E |
| Entognatha | Collembola | Entomobryidae | | sp.6 | X | | E |
| Entognatha | Collembola | Isotomidae | | sp.1 | X | X | E/P |
| Entognatha | Collembola | Paronellidae | | sp.1 | X | X | E/P |
| Entognatha | Collembola | Sminthuroidea | | sp.2 | X | X | E/P |
| Entognatha | Diplura | Campodeidae | | sp.1 | X | X | E/P |
| Entognatha | Diplura | Japygidae | | sp.1 | | X | P |
| Entognatha | Diplura | Japygidae | | sp.3 | | X | E |
| Insecta | Archaeognatha | Meinertellidae | | sp.1 | X | | E |
| Insecta | Blattodea | Blaberidae | | sp.1 | 6 | 5 | E/P |
| Insecta | Blattodea | Blaberidae | | sp.2 | 8 | 1 | P |
| Insecta | Blattodea | Blaberidae | | sp.3 | | 1 | P |
| Insecta | Blattodea | Blattellidae | | sp.1 | 14 | 10 | E/P |
| Insecta | Blattodea | Blattellidae | | sp.5 | 6 | 3 | P |
| Insecta | Coleoptera | Carabidae | | sp.11 | X | | E |
| Insecta | Coleoptera | Chrysomelidae | | sp.4 | X | | P |
| Insecta | Coleoptera | Dytiscidae | | sp.7 | | X | E |
| Insecta | Coleoptera | Dytiscidae | | sp.12B | X | X | P |
| Insecta | Coleoptera | Histeridae | | sp.1 | | X | P |
| Insecta | Coleoptera | Ptiliidae | | sp.1 | | X | P |
| Insecta | Coleoptera | Staphylinidae | Pselaphinae | sp.1 | | X | E |
| Insecta | Coleoptera | Staphylinidae | Pselaphinae | sp.11 | X | | E |
| Insecta | Coleoptera | Staphylinidae | Pselaphinae | sp.16 | X | | P |
| Insecta | Coleoptera | Staphylinidae | | sp.10 | | X | P |
| Insecta | Diptera | Cecidomyiidae | Cecidomyiinae | sp. | X | X | E/P |
| Insecta | Diptera | Ceratopogonidae | | sp. | X | | P |
| Insecta | Diptera | Chironomidae | | sp. | | X | E |
| Insecta | Diptera | Chloropidae | | sp. | X | | P |
| Insecta | Diptera | Culicidae | *Culex* | sp. | X | X | E |
| Insecta | Diptera | Dolichopodidae | | sp. | X | | E |

| | | | | | | | |
|---|---|---|---|---|---|---|---|
| Insecta | Diptera | Ephydridae | | sp. | | X | E |
| Insecta | Diptera | Milichiidae | *Milichiella* | sp. | | X | E/P |
| Insecta | Diptera | Muscomorpha | | sp. | X | | P |
| Insecta | Diptera | Phoridae | Metopininae | sp. | X | | E |
| Insecta | Diptera | Psychodidae | Phlebotominae | sp. | X | X | E/P |
| Insecta | Diptera | Sciaridae | | sp. | X | X | E/P |
| Insecta | Diptera | Streblidae | *Trichobius* | sp. | | X | P |
| Insecta | Diptera | Tipulidae | Tipulinae | sp. | X | X | E/P |
| Insecta | Heteroptera | Cydnidae | | sp. | X | X | E/P |
| Insecta | Heteroptera | Dipsocoroidea | | sp. | X | X | E/P |
| Insecta | Heteroptera | Reduviidae | Emesinae | sp. | 2 | 2 | E/P |
| Insecta | Heteroptera | Reduviidae | *Panstrongylus* | jovem | 2 | | P |
| Insecta | Heteroptera | Reduviidae | Reduviinae | sp. | 6 | 7 | E |
| Insecta | Heteroptera | Reduviidae | Triatominae | sp. | | 1 | |
| Insecta | Homoptera | Cixiidae | | sp.5 | | X | E |
| Insecta | Homoptera | Cixiidae | | sp.7 | X | | E |
| Insecta | Hymenoptera | Apoidea | | sp.1 | 1 | | E |
| Insecta | Hymenoptera | Braconidae | | sp.1 | | X | E |
| Insecta | Hymenoptera | Diapriidae | | sp.2 | | X | P |
| Insecta | Hymenoptera | Formicidae | *Camponotus* | sp.1 | X | | E |
| Insecta | Hymenoptera | Formicidae | *Camponotus* | sp.2 | X | X | E/P |
| Insecta | Hymenoptera | Formicidae | *Crematogaster* | sp.1 | X | X | E |
| Insecta | Hymenoptera | Formicidae | *Dolichoderus* | aff. *bispinosus* | X | X | E/P |
| Insecta | Hymenoptera | Formicidae | *Eciton* | sp.2 | | X | E |
| Insecta | Hymenoptera | Formicidae | *Eurhopalothrix* | sp.1 | X | X | E/P |
| Insecta | Hymenoptera | Formicidae | *Gnamptogenys* | sp.1 | X | | E/P |
| Insecta | Hymenoptera | Formicidae | *Hypoponera* | sp.2 | X | X | E |
| Insecta | Hymenoptera | Formicidae | *Labidus* | sp.1 | X | | E/P |
| Insecta | Hymenoptera | Formicidae | *Pachycondyla* | sp.1 | | X | E |
| Insecta | Hymenoptera | Formicidae | *Pachycondyla* | sp.2 | X | X | E/P |
| Insecta | Hymenoptera | Formicidae | *Paratrechina* | sp.1 | | X | E |
| Insecta | Hymenoptera | Formicidae | *Pheidole* | sp.1 | X | X | E/P |
| Insecta | Hymenoptera | Formicidae | *Pheidole* | sp.2 | X | X | E |
| Insecta | Hymenoptera | Formicidae | *Pheidole* | sp.3 | X | X | E |
| Insecta | Hymenoptera | Formicidae | *Solenopsis* | sp.1 | X | X | E/P |
| Insecta | Hymenoptera | Formicidae | *Solenopsis* | sp.2 | X | X | E/P |
| Insecta | Hymenoptera | Formicidae | *Trachymyrmex* | sp.1 | | X | |
| Insecta | Hymenoptera | Vespidae | | sp.1 | | X | E |
| Insecta | Isoptera | Termitidae | *Convexitermes* | sp. | X | | E |
| Insecta | Isoptera | Termitidae | *Diversitermes* | sp. | X | | E |
| Insecta | Isoptera | Termitidae | *Embiratermes* | sp. | X | X | E |
| Insecta | Isoptera | Termitidae | *Nasutitermes* | sp. | X | X | E/P |
| Insecta | Isoptera | Termitidae | *Neocapritermes* | sp. | | X | E |
| Insecta | Lepidoptera | Gelechioidea | | sp. | X | X | E |
| Insecta | Lepidoptera | Noctuoidea | | sp.1 | 1 | 6 | E |
| Insecta | Lepidoptera | Noctuoidea | | sp.5 | 2 | | E |
| Insecta | Lepidoptera | Tineoidea | | sp.1 | | X | E |
| Insecta | Lepidoptera | Tineoidea | | sp.2 | X | X | P |
| Insecta | Lepidoptera | Tineoidea | | sp.3 | X | X | E/P |
| Insecta | Mantodea | Mantidae | | sp.1 | 1 | | |
| Insecta | Neuroptera | Myrmeleontidae | | jovem | X | | E |
| Insecta | Orthoptera | Phalangopsidae | *Paraclodes* | sp. | 24 | 39 | E |

| | | | | | | | |
|---|---|---|---|---|---|---|---|
| Insecta | Orthoptera | Phalangopsidae | *Phalangopsis* | sp. | 505 | 255 | E/P |
| Insecta | Plecoptera | | | jovem | | 1 | E |
| Insecta | Psocoptera | Epipsocidae | *Epipsocus* | jovem | X | | P |
| Insecta | Psocoptera | Epipsocidae | *Mesepipsocus* | sp.1 | | X | E |
| Insecta | Psocoptera | Psyllipsocidae | *Psyllipsocus* | sp.1 | X | | E |
| Insecta | Siphonaptera | Leptopsyllidae | | sp.1 | X | | P |
| Insecta | Siphonaptera | Rhopalopsyllidae | | sp.1 | | X | P |
| Insecta | Thysanoptera | Thripidae | | sp.1 | X | | E |
| Insecta | Thysanura | Nicoletiidae | | sp.1 | X | X | E/P |
| Gastropoda | Pulmonata | Subulinidae | | sp.2 | X | X | P |
| Gastropoda | Pulmonata | Systrophiidae | *Happia* | sp. | X | X | E/P |
| Malacostraca | Isopoda | Armadillidae | | sp.1 | X | X | E/P |
| Malacostraca | Isopoda | Philosciidae | | sp.1 | X | X | E/P |
| Malacostraca | Isopoda | Scleropactidae | *Circoniscus* | *buckupi* | X | X | P |
| Malacostraca | Isopoda | Scleropactidae | *Circoniscus* | sp.2 | | X | E |
| Onychophora | Onychophora | Peripatidae | *Peripatus* | sp.1 | 1 | | |
| Symphyla | | Scutigerellidae | | sp. | X | X | E |
| Amphibia | Anura | | | sp. | 24 | 9 | E/P |
| Mammalia | Chiroptera | Emballonuridae | *Peropteryx* | *kappleri* | 2 | 1 | E |
| Mammalia | Chiroptera | Furipteridae | *Furipterus* | *horrens* | | 1 | P |
| Mammalia | Chiroptera | Phyllostomidae | *Carollia* | *perspicillata* | 200 | 200 | E/P |
| Mammalia | Chiroptera | Phyllostomidae | *Desmodus* | *rotundus* | 1 | | P |
| Mammalia | Chiroptera | Phyllostomidae | *Diphylla* | *ecaudata* | | 1 | P |
| Mammalia | Chiroptera | Phyllostomidae | *Lonchorrhina* | *aurita* | 100 | 15 | E/P |
| Mammalia | Chiroptera | Phyllostomidae | *Trachops* | *cirrhosus* | 6 | 10 | E/P |
| Mammalia | Rodentia | Cricetidae | *Rhipidomys* | sp. | | 1 | P |
| Reptilia | Squamata | Gekkonidae | *Thecadactylus* | sp. | | 1 | E |
